# Bile acid diarrhoea and metabolic changes after cholecystectomy: a prospective case-control study

**DOI:** 10.1186/s12876-024-03368-8

**Published:** 2024-08-22

**Authors:** Alexia Farrugia, Nigel Williams, Saboor Khan, Ramesh P. Arasaradnam

**Affiliations:** 1 Department of Surgery, Sandwell and West Birmingham NHS Trusts, Birmingham, UK; 2 Department of Surgery, University Hospitals Coventry and Warwickshire, Coventry, UK; 3 Department of Gastroenterology, University Hospitals Coventry and Warwickshire, Coventry, UK

**Keywords:** Diarrhoea, Cholecystectomy, Bile acids

## Abstract

**Introduction:**

Bile acid diarrhoea (BAD) can occur due to disruption to the enterohepatic circulation such as following cholecystectomy. However, the mechanism behind this is as yet unknown. The aim of this study was to determine the rate of post-cholecystectomy diarrhoea and to assess whether FGF19 within the gallbladder was associated with the development of BAD.

**Methods:**

This was a prospective case-control study in which patients were assessed pre- and post- cholecystectomy (study group) and compared with patients also having laparoscopic surgery but not cholecystectomy (control group). Their bowel habits and a GIQLI questionnaire was performed to compare the pre- and post-operative condition of the two groups. Gallbladder tissue sample was tested for FGF19 and PPARα in the study group patients. A subset had serum lipid levels, FGF19 and C4 measurements.

**Results:**

Gallbladder PPAR α was found to have a significant correlation with stool consistency, with the lower the PPARα concentration the higher the Bristol stool chart number (i.e. looser stool). There were no significant correlation when assessing the effect of gallbladder FGF19 concentration on bowel habit, stool consistency, lipid levels, BMI or smoking. The study group showed a significant increase in triglycerides post-operatively, however there were no changes in cholesterol, HDL and LDL levels. Correlation of the increased triglyceride levels with stool consistency and frequency showed no significant results

**Discussion and conclusion:**

We did not find any direct evidence that FGF19 levels within the gallbladder impact the development of post-cholecystectomy diarrhoea. There was however a significant increase in triglycerides postoperatively. There was also no correlation of bowel habits with PPARα suggesting the observed rise is independent of this pathway. Further work is required particularly relating to the gut microbiome to further investigate this condition.

## Background

The role of bile acids (BA) within the body is to aid in digestion of fatty acids, where they are released into the duodenum after stimulation of the gallbladder by cholecystokinin. They are then reabsorbed from the terminal ileum. This reabsorption is controlled by two negative feedback loops in which fibroblast growth factor 19 (FGF19) is an essential factor in inhibiting bile acid synthesis [1]. In one loop FGF19 inhibits bile acid synthesis by activating Farnesoid X receptor (FXR), and in the other loop it binds Fibroblast Growth Factor Receptor 4 (FGFR4) in the hepatocytes. Both lead to inhibition of cholesterol 7 alpha hydroxylase enzyme (CYP7A1), which is the rate limiting step in bile acid synthesis [1, 2]. Interruption of these feedback loops, or overwhelming them, can cause bile acid diarrhoea (BAD), which could be either primary or secondary. Primary bile acid diarrhoea is idiopathic while secondary tends to occur after other conditions such as cholecystectomy [3]. When the negative feedback mechanism is disrupted, as occurs in bile acid diarrhoea, the activity of CYP7A1 is increased and there is a six- to seven-fold increase in the synthesis of bile acids [4]. The absorptive capacity of the terminal ileum is exceeded due to large amounts of bile acids and therefore this leads to diarrhoea [5].

The ^75^SeHCAT test, which is used to measure bile acid retention, is the gold standard for diagnosing BAD. A value of < 15% retention is indicative of BAD [5]. However, it is often impractical, therefore other diagnostic tools for BAD are used, which include measurement of serum C4. This is a direct measure of bile acid synthesis and is increased in BAD, therefore it can be used in cases of ^75^SEHCAT unavailability [6].

Hypertriglyceridaemia has been linked to increased bile acid synthesis and higher triglyceride levels are associated with lower ^75^SeHCAT retention levels. It has been demonstrated that primary bile acid diarrhoea was significantly associated with higher triglyceride levels [7]. Post-cholecystectomy there is faster circulation of BA, resulting in negative feedback and therefore lower triglyceride levels [8, 9]. However, there is conflicting information as some studies show no change in lipid levels, including any difference between patients who develop post cholecystectomy diarrhoea and those who do not [10].

Lower FGF 19 levels, which normally occurs in BAD, causes inhibition of short heterodimer primer (SHP) which leads to higher triglyceride levels and therefore increased bile acid synthesis, leading to increased low-density lipoprotein (LDL) uptake. Lack of FXR activation also means that LDL activity is also increased, and these factors work together resulting in hypertriglyceridaemia [11]. Patients with hypertiglyceridaemia have impaired intestinal BA absorption due to reduced expression of apical sodium-dependent bile acid transporter (ASBT) which is involved in BA uptake, therefore leading to reduced FGF19 levels and reduced negative feedback on bile acid synthesis [12]. Peroxisome proliferator-activated receptor alpha (PPARα) decreases triglyceride levels by causing free fatty acid oxidation and is activated by increased FXR levels. Thus, in BAD where FXR is not activated there is lack of PPARα activation and patients have hypertriglyceridaemia.

The aims of the study were to determine the frequency of bile acid diarrhoea and whether there is a change in bowel habit, including stool consistency after laparoscopic cholecystectomy and if there was any correlation to any metabolic changes, including gallbladder FGF19 or SHP and serum C4 and FGF19. The secondary aims were to determine the change in lipid levels (LDL, HDL and triglycerides) post-cholecystectomy and the mechanism behind this change along with its relationship to the development of bile acid diarrhoea, and whether gallbladder PPARα is associated with any change in lipid levels.

## Methods

Approval was gained from the ethics committee and the Health research authority for a case control study comparing two groups (Ref 18/EM/0395). This was a prospective study and the study period was from September 2019 to March 2020. The study was performed at University Hospitals Coventry and Warwickshire. The study group consisted of those undergoing laparoscopic cholecystectomy. The age-matched control group also had diagnostic laparoscopic surgery. These were mainly patients undergoing laparoscopic Nissen fundoplications, laparoscopic hernia repair, and laparoscopic bariatric surgery. All patients who were having cancer surgery were excluded, as well as those under the age of 18.

Stool frequency and consistency (using the Bristol stool chart) were recorded at the pre-operative stage as well as three months postoperatively. They were also given the option to have blood tests taken for measurement of lipid levels (control and study group), C4 and FGF19 (study group only) again before and three months after surgery. 11 patients from the study group and 9 patients from the control group took this option. Those patients having cholecystectomy were also asked for a gallbladder sample when it was removed. All patients in the study group took this option. The gallbladder tissue was tested for FGF19 and PPARα via ELISA (enzyme-linked immunoabsorbent assay) testing. Anyone who developed diarrhoea as per the British Society of Gastroenterology (BSG) criteria (which is defined as persisting alteration from normal stool, with a Bristol stool type form 5 to 7, for more than 4 weeks), was offered a ^75^SeHCAT scan and a colonoscopy with ileal biopsy [13].

A sample size of 110 was determined using a power calculation based on post-cholecystectomy diarrhoea rates from previous studies. Unfortunately, the study had to be stopped prior to reaching the sample size due to service re-distribution during the COVID pandemic. Age, sex and BMI of the two groups were compared using a Chi-squared test. Differences in lipid levels between the groups were assessed using a Wilcoxon signed rank test. Correlations between lipid levels, C4 levels and FGF19 levels and bowel habit and GIQLI questionnaire scores were performed using a Spearman’s correlation coefficient. IBM SPSS statistical software version 23 was used to perform statistical analysis and GraphPad PRISM version 10.1.0 was used for graphics.

## Results

The two groups were analysed for demographic differences. There were no significant differences in the age, sex and BMI between the two groups, *p* = 0.316, *p* = 0.094 and *p* = 0.279 respectively, using a Chi-squared test. These are shown in Table 1 below. Of the 40 patients in the study group, 36 were followed up and four developed BAD (11.1%), diagnosed via ^75^SeHCAT scan.

**Table 1:** Demographic data

	Study group	Control group	*p*-value
Total	40	20	-
Male: Female	11:29	11:9	*p* = 0.94
Median BMI (range)	28.5(20-41.8)	32.7 (22–54)	*p* = 0.297
Median age (range)	48.5 (20–76)	52 (32–76)	*p* = 0.316
Smoker: ex-smoker: non-smoker	8:13:19	1:3:16	*p* = 0.1
Comorbidities • GORD • IBS/colitis	24 • 5 • 2	13 • 3 • 0	*P* = 0.1

### Metabolic parameters

**Fig. 1:**
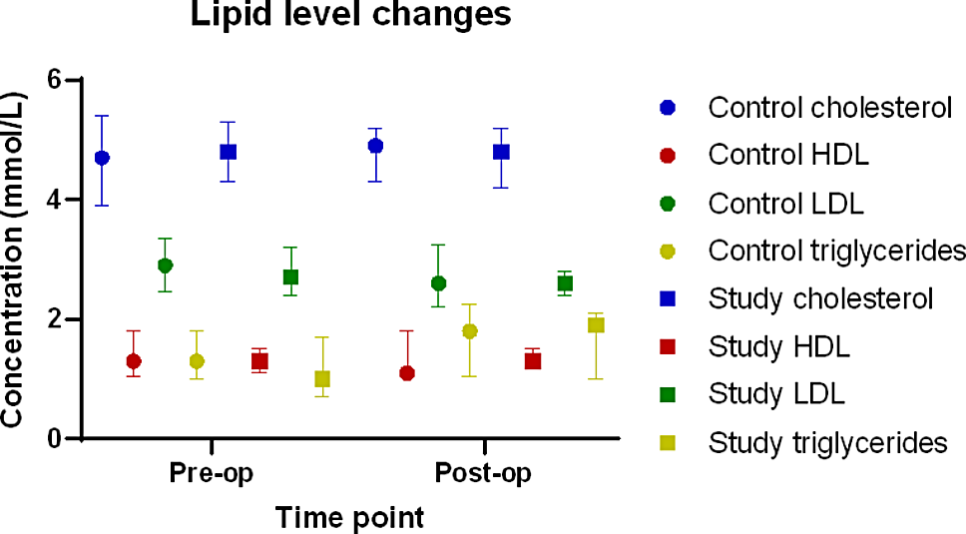
Lipid levels, study group, expressed as median and IQR

There were no significant differences between pre- and post-op level of cholesterol (*p* = 0.812) HDL (*p* = 0.944), and LDL (*p* = 0.082). There was a significant difference in pre- and post- operative triglyceride levels (*p* = 0.021). Triglyceride levels were significantly increased post operatively, as the mean (+/- confidence interval) pre-op was 1.25 (+/- 0.4) mmol/L and the average (+/- confidence interval) post-op was 2.07 (+/- 1.1) mmol/L. There were no significant differences in cholesterol, HDL, triglyceride or LDL levels in the pre- and post-operative period for the control group. These are shown in Fig. 1.

**Fig. 2:**
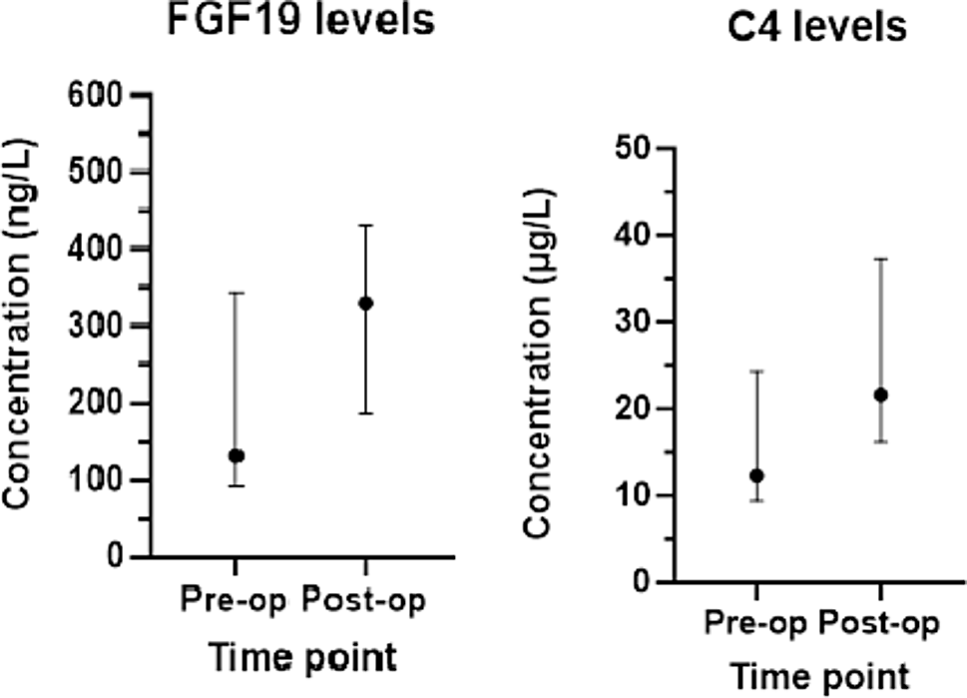
Plasma FGF19 and C4 levels, expressed as median and IQR

There is a general trend in increasing FGF19 plasma concentration levels postoperatively. There was found to be a significant difference between the pre- and postoperative fasting plasma FGF19 levels (*p* = 0.043), with the levels being significantly higher post op. There was no correlation between the change that occurred in plasma FGF19 levels and change in stool consistency (*p* = 0.40), change in bowel habit (*p* = 0.99) or change in GIQLI scores (*p* = 0.1). There were no significant differences in the pre- and post-op C4 levels (*p* = 0.18). There was also no correlation between change in C4 levels and change in bowel habit, stool consistency and GIQLI results (*p* = 0.72, *p* = 0.23, and *p* = 0.071 respectively). These are seen in Fig. 2.

### Gallbladder tissue

**Fig. 3:**
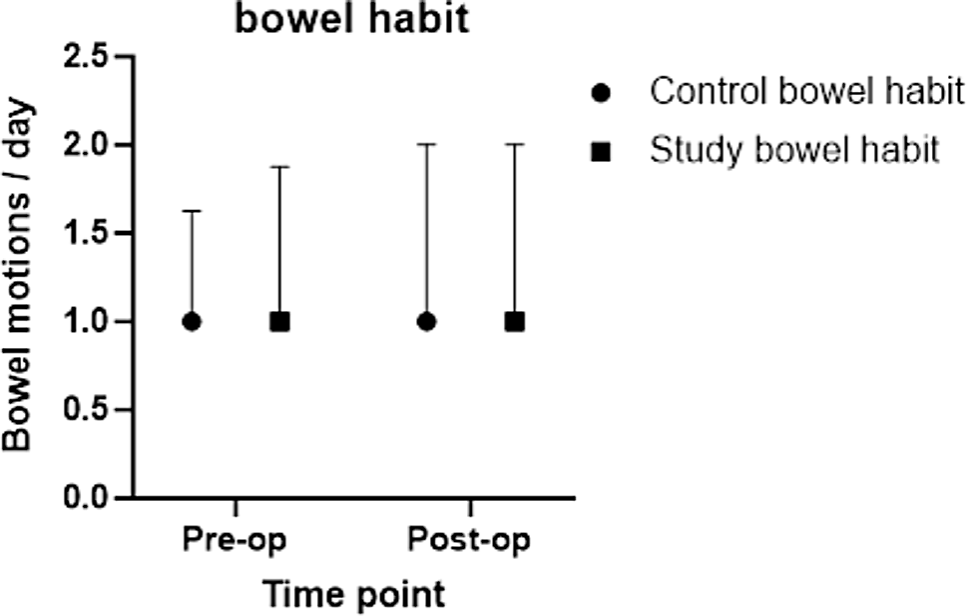
Bowel habits, expressed as median and IQR

We tested for correlation between FGF19 concentration in gallbladder tissue and change in bowel habit, stool consistency, BMI and smoking status. These tests revealed no significant correlation (*p* = 0.124, *p* = 0.173, *p* = 0.424 and *p* = 0.523 respectively). The mean concentration of FGF 19 in pg/ml was also correlated to the change in triglyceride levels. There was no significant correlation between the two variables (*p* = 0.581). Analysis of whether there was a relationship between the change in plasma FGF19 levels and the gallbladder FGF19 concentration was performed and found to be negative (*p* = 0.65). They are shown in Fig. 3.

We also tested for correlation between PPARα concentration and change in bowel habit and triglyceride levels and again there was no significant correlation (*p* = 0.12 and *p* = 0.748 respectively). However, there was a significant correlation with change in stool consistency (*p* = 0.003), showing a lower concentration with higher Bristol stool chart value (looser stool).

## Discussion

When looking at metabolic parameters, an interesting point to note was that the FGF19 levels were significantly higher postoperatively and there is a generally increasing trend of FGF19 levels in plasma postoperatively. The increased concentration of FGF19 could be due to possible negative feedback post-gallbladder removal, as there is no longer a ‘storage system’ for bile acids. Thus, these are continuously being released into the small bowel and triggering more FGF19 transcription in the terminal ileum. It would be interesting to see whether this would be consistent with a larger cohort. There has been similar published data which found a significant difference post-operatively without the patients being given a meal, however there has also been a similar study with 18 patients which did not find any difference in the FGF19 levels post-cholecystectomy initially which changed once patients were given a meal [14].

When analysing C4 levels, there were no significant correlations to bowel habit, stool consistency and GIQLI results. There were also no significant differences in C4 levels pre- and post-op. This is interesting as C4 levels would be expected to be higher post-operatively to reflect increased bile acid production, as has been previously reported [10]. However, in Sauter’s data the increase in levels was not statistically significant so it is difficult to assess. They also did not report any changes associated with bowel habits. The study by Borup et al. also did not find any significant differences in pre- and post- operative C4 levels [14].

The overall increased FGF19 levels would imply a reduction in bile acid synthesis via a negative feedback loop, with FGF19 acting on FXR within the liver [3]. However, the increased C4 levels would actually indicate an increase in bile acid synthesis [6]. As there were no significant differences in stool frequency and consistency within the study group it may be that the increase in FGF19 was not enough to effect a change in the bowel habit of this population. The increase in C4 level may be explained by the increase in enterohepatic cycles post-removal of the gallbladder. FGF19 also has a diurnal variation and therefore it may be that this may have been affected by cholecystectomy [15].

While FGF19 is present within the gallbladder, its function within this organ is unknown. We postulated that since FGF19 is also secreted by the gallbladder, a higher FGF19 concentration in the gallbladder may result in the development of bile acid diarrhoea once it is removed due to a potential role in this negative feedback loop [16]. However, there were no significant correlations between FGF19 concentration within the gallbladder and the changes in bowel habits exhibited by the patients. This may imply either that the FGF19 level secreted by the gallbladder are not high enough to be an effective part of the negative feedback loop, or that the feedback loop is interrupted at a level downstream from FGF19 when bile acid diarrhoea develops. It may also imply that the FGF 19 from the gallbladder is not related to the negative feedback loop at all. This may explain also why there is no correlation between the plasma FGF19 concentration levels and gallbladder FGF19 concentration levels.

PPARα is involved in the regulation of lipid levels by regulating fatty acid metabolism once it is activated by FXR. It decreases hepatic apo C-III production and increases LPL-mediated lipolysis which then increases triglyceride metabolism and decreases LDL secretion. This causes increased free fatty acid oxidation and decreasing serum triglyceride levels [1, 17]. Thus, we investigated the effect of gallbladder PPARα on bowel habits as well as on triglyceride levels. We have shown that there was no correlation of PPARα concentration within the gallbladder with the change in triglyceride levels post-operatively. However, when taking the whole picture into account, there was a significant correlation between PPARα concentration levels and change in stool consistency postoperatively, though this was not reflected in the change in bowel habits. This correlation with change in stool consistency may be a reflection of the interruption of the bile acid synthesis loop where there are higher FXR levels leading to more PPARα activation, with the interruption of the negative feedback loop coming later in the pathway thus leading to higher bile acid synthesis rates (rather than lower synthesis rates as it should be with higher FXR concentrations).

This was an exploratory mechanistic study to show metabolic changes post-cholecystectomy. The control group does have some drawbacks in that there may have been some other metabolic changes, however we have shown that PPARα levels do not drive triglyceride levels. We have also shown that serum FGF19 levels change in the post-cholecystectomy period however more work needs to be done regarding the mechanism. The limitations of this study include that it was a single-centre study with a small sample size and therefore it is exploratory in nature.

## Conclusion

We have performed the first mechanistic human study investigating the pathophysiology of bile acid diarrhoea following cholecystectomy. We have found that FGF19 levels within the gallbladder have no known effect on the development of BAD, however have confirmed that plasma FGF19 increases after surgery, as do triglyceride levels. We also found that lower gallbladder PPARα concentrations were associated with looser stool which may also be related to the interruption of the negative feedback loop. This study may form the basis of multiple larger studies in the future, including those investigating the gut microbiome and relationship with cholecystectomy and the role of biomarkers such as FGF19 or PPARα in the development of BAD.

## Data Availability

No datasets were generated or analysed during the current study.
